# Psychological distress in stroke patients: latent class analysis and influencing factors

**DOI:** 10.3389/fpsyt.2026.1882641

**Published:** 2026-08-07

**Authors:** Lingjun Luo, Yunmei Guo, Weiyan Tian, Linfei Li, Xianjuan Gou

**Affiliations:** Department of Emergency, Affiliated Hospital of Zunyi Medical University, Zunyi, Guizhou, China

**Keywords:** health literacy, latent class analysis, psychological distress, SCL-90, stroke

## Abstract

**Background:**

Psychological distress is common in stroke patients and adversely affects rehabilitation and quality of life. However, the heterogeneity of this distress remains underexplored.

**Aim:**

This study aimed to explore the latent categories of psychological distress in patients with stroke and analyze its influencing factors.

**Methods:**

A cross-sectional study was conducted using a convenience sample of 417 patients with stroke in a tertiary hospital in Zunyi from July to November 2024. Socio-demographic information and psychological distress of participants were obtained using a general information questionnaire and the SCL-90 scale. Latent class analysis was used to analyze the latent categories of psychological distress in patients with stroke. In the multiple logistic regression analysis, the latent categories of psychological distress derived from latent class analysis were used as the dependent variable to identify factors influencing these categories.

**Results:**

Psychological distress in patients with stroke was classified into three groups: High Paranoid Ideation and Hostility group (Class 1, 17.99%), Generalized Psychological Distress group (Class 2, 25.18%), and Low Psychological Distress group (Class 3, 56.83%). Multiple logistic regression demonstrated that, compared with Class 3 (reference), health literacy was significantly associated with membership in Class 1 (OR = 1.019, 95% CI: 1.003–1.036) and Class 2 (OR = 1.057, 95% CI: 1.039–1.075), while interoceptive sensibility was significantly associated with membership in Class 1 (OR = 0.988, 95% CI: 0.980–0.997) and Class 2 (OR = 0.974, 95% CI: 0.965–0.983). Male patients and those with a stroke duration of less than one year were significantly more likely to belong to Class 2 (OR = 9.281, 95% CI: 4.347–19.815; OR = 23.594, 95% CI: 2.592–214.62).

**Conclusion:**

Among stroke patients, psychological distress manifests in three distinct profiles: the High Paranoid Ideation and Hostility group, the generalized psychological distress group, and the low psychological distress group. Tailored intervention measures are implemented based on the unique demographic characteristics of each group to enhance their mental health outcomes.

## 1 Background

Stroke, also known as cerebrovascular accident or cerebral infarction, is a general term for a group of diseases caused by cerebrovascular lesions that result in impaired brain function. According to data from the 2021 Global Burden of Disease Study, there are currently approximately 101 million stroke patients worldwide (1). In a large, nationally representative sample of adults aged 40 years or older, the estimated prevalence, incidence, and mortality rate of stroke in China in 2020 were 2.6%, 505.2 per 100–000 person-years, and 343.4 per 100–000 person-years, respectively (2, 3). Following a stroke, approximately 60%–80% of patients will experience varying degrees of functional impairment. Due to factors such as disability and disrupted self-image, patients may experience significant psychological distress during the rehabilitation process (4–6). Research indicates that in Western developed countries, approximately one-third of stroke survivors experience psychological distress shortly after the onset of the disease (7, 8).

Psychological distress is a common mental health issue, also known as emotional distress, psychological stress, or psychological pain, and refers to unpleasant emotional experiences caused by multiple factors (9). It is a sub-health state between normal psychology and mental illness, involving psychological, social, and spiritual dimensions. Psychological distress has negative effects on the treatment and rehabilitation of stroke patients, reducing the long-term effectiveness of functional exercises and quality of life, and even increasing the risk of stroke recurrence and death (10).

Despite its clinical importance, current research on psychological distress in stroke patients has largely adopted variable-centered approaches (e.g., defining cutoff values for total scale scores), which assume homogeneity within the patient population and fail to adequately recognize individual heterogeneity (11). However, stroke patients may exhibit diverse psychological response patterns, and understanding this heterogeneity is critical for developing targeted interventions.

Latent Class Analysis (LCA) is a multivariate statistical model that estimates the probability of each participant being assigned to each subgroup using maximum likelihood estimation and assigns each participant to the group with the highest probability (12). This method focuses on the heterogeneity of psychological distress in stroke patients, categorizing psychological distress into subtypes with specific characteristics. It not only describes quantitative differences between individuals but also summarizes qualitative differences between groups. Recent studies have begun to apply LCA to examine symptom heterogeneity in stroke populations. Chen et al. (11). identified distinct trajectories of depression-related symptom clusters, Dong et al. (13) explored psychoneurological symptom clusters in acute stroke, Chen et al. (14) examined depression-anxiety comorbidity in ischemic stroke patients, Chen et al. (15) classified psychosomatic symptom clusters in middle-aged and older survivors, Huang et al. (16) identified depression-fatigue-pain classes by sex, Han et al. (17) characterized early-onset PSD profiles, and Cao et al. (18) examined physical-mental symptoms during recovery. Additionally, our group has published a protocol for a longitudinal LCA study on psychological distress in stroke patients (19). Despite these advances, no study has specifically applied LCA to comprehensively characterize psychological distress profiles using all nine SCL-90 dimensions in a general stroke population, nor has any study examined health literacy and interoceptive sensibility simultaneously as influencing factors.

Therefore, this study aimed to: (1) identify distinct latent classes of psychological distress among stroke patients using LCA, and (2) examine the factors associated with class membership, including health literacy, interoceptive sensibility, and demographic/clinical characteristics.

## 2 Methods

### 2.1 Design and data collection

This research is a cross-sectional observational study. It involved recruiting stroke patients via convenience sampling from the Department of Cerebral Vessels at the Affiliated Hospital of Zunyi Medical University in Zunyi City, Guizhou Province, China, between July and November 2024. This study followed the Strengthening the Reporting of Observational Studies in Epidemiology (STROBE) guidelines for cross-sectional studies.

The inclusion criteria consisted of the following: (1) a confirmed diagnosis of ischemic or hemorrhagic stroke verified by computed tomography or magnetic resonance imaging (20); (2) an age of 18 years or older; (3) the ability to communicate effectively and comprehend simple instructions; and (4) the provision of informed consent. The exclusion criteria included: (1) the presence of unconsciousness or mental disorders; and (2) the presence of severe heart failure, respiratory failure, tumors, or other terminal conditions.

Two researchers who had undergone uniform training screened potential participants in the Department of Cerebral Vessels and determined the final study cohort based on the inclusion and exclusion criteria. Initially, one researcher led the subjects in groups to a quiet classroom to facilitate conversation and data collection. Subsequently, another researcher individually introduced and explained the study’s main objectives, significance, and procedures. They assured participants of privacy protection and obtained their consent to participate by signing an informed consent form.

### 2.2 Instruments

Demographic questionnaire. A checklist included questions on the following demographic features: age, gender, residence, ethnicity, educational level, marital status, living conditions, household income, and disease duration.

### 2.3 Psychological distress

The Symptom Checklist-90 (SCL-90) was utilized to assess the psychological distress among patients (21, 22). This instrument comprises 90 items, covering various dimensions such as somatization, obsessive-compulsive symptoms, interpersonal sensitivity, depression, anxiety, hostility, phobic anxiety, paranoid ideation, psychoticism, and other related factors. The scoring method follows a 5-point scale, where 1=‘none,’ 2=‘mild,’ 3=‘moderate,’ 4=‘severe,’ and 5=‘extreme.’ A total score of 160 or any factor score of 2 or higher may indicate significant psychological distress. The Cronbach’s coefficient of the SCL-90 in this study was 0.97.

### 2.4 Interoceptive sensibility

The Multidimensional Assessment of Interoceptive Awareness, Version 2 (MAIA-2) was employed to evaluate interoceptive sensibility (23). This 37-item scale is structured around eight factors: noticing, distracting, worrying, attention regulation, emotional awareness, self-regulation, body listening, and trust. In its initial psychometric analysis, the internal consistency reliability varied from 0.64 for noticing to 0.83 for attention regulation and trust. MAIA-2 scores are determined on a 0–5 scale, with higher values representing greater levels of the assessed trait. Our scoring method for the distracting and worrying domains aligns with their direct meaning, rather than using the reverse-scoring approach sometimes applied to these two domains (i.e., we maintained their original scoring format instead of reversing it).

### 2.5 Health literacy scale

The study utilized the Chinese adaptation of the Health Literacy Management Scale (HeLMS) to assess patients’ health literacy (HL) (24). This scale comprises 24 items across four dimensions: communication and interaction ability (9 items, max score 45), information acquisition ability (9 items, max score 45), motivation to enhance health (4 items, max score 20), and financial commitment to health (2 items, max score 10). Each item is scored on a 5-point scale from 1 (very difficult) to 5 (not at all difficult), with higher scores signifying greater HL proficiency. The maximum total score achievable is 120 points.

### 2.6 Data analysis

In this study, SPSS 28.0 and Mplus 8.3 statistical software were used for data analysis, with a significance level of P<0.05 for statistical tests. Categorical data were described using frequencies (proportions), while measurement data were characterized by means and standard deviations.

Mplus 8.3 was used to explore latent classes of psychological distress in stroke patients, scored on a Likert 5-point scale. The nine dimension scores of the SCL-90 were used as indicators. Scores ≥2 were coded as 1 (positive response), and <2 as 0 (negative response), following the established SCL-90 clinical criterion. Although dichotomization involves some information loss, it is widely used in LCA studies for clinically meaningful classification. These transformed scores from each dimension served as manifest indicators in the statistical model. Starting with a one-class model, the number of latent classes was incrementally increased. The best-fitting model was selected by comparing fit indices, including Log likelihood, Akaike Information Criterion (AIC), Bayesian Information Criterion (BIC), sample-size adjusted BIC (aBIC), Entropy, Lo-Mendell-Rubin (LMR) test, and Bootstrapped Likelihood Ratio Test (BLRT). Lower AIC, BIC, and aBIC values indicated better fit, while Entropy values closer to 1 indicated higher classification accuracy. LMR and BLRT assessed fit differences between consecutive models, with P<0.05 suggesting a significantly better k-class model over a (k-1)-class model (25). The optimal model was chosen based on model fit, theoretical relevance, and interpretability of results.

This study used multivariate logistic regression to identify factors influencing the latent classes of psychological distress in stroke patients. First, univariate logistic regression analyses were conducted for each independent variable to screen for those with a statistical association (P<0.1). These variables were then included in a multivariate model, using stepwise regression to determine significant independent factors. Results are presented as odds ratios (OR) and 95% confidence intervals (CI).

## 3 Results

### 3.1 Participant demographic characteristics

**Table 1** presents the demographic characteristics of 417 stroke patients. The mean age is 61.75 years. Most participants are male (62.4%), live in the countryside (89.2%), and are Han-Nationality (92.8%). The majority have elementary education (70.74%), are married (95.9%), and live with their spouse (77.7%). Regarding income, 58.99% earn a moderate income, and 11.51% have a high income. Ischemic stroke is the most common type (68.8%), and 65.2% require assistance. The duration of stroke is less than 1 year for 75.54% of patients. Interoceptive sensitivity is 90.46 ± 44.92, and health literacy is 69.33 ± 22.82.

**Table 1:** Demographic characteristics of participants (n=417).

Variable	Categories	Mean (SD)/frequency (N)	Percentage(%)
Age		61.75 ± 12.26	
Gender	Male	260	62.40
	Female	157	37.60
Residence	Countryside	372	89.20
	City	45	10.80
Ethnic group	Han-Nationality	387	92.80
	Ethnic minority	30	7.20
Education	Elementary	295	70.74
	Middle school	88	21.10
	High school	22	5.28
	College	12	2.88
Marital status	Married	400	95.90
	Widowed/divorced	17	4.10
Living Situation	Living alone	22	5.28
	Living with spouse	324	77.70
	Living with children or grandchildren	71	17.03
Family monthly income	< 1,500 RMB	123	29.50
	1,500–3,000 RMB	246	58.99
	> 3,000 RMB	48	11.51
Types of stroke	Ischemic stroke	287	68.80
	Hemorrhagic stroke	130	31.20
Self-care capability	Independent	145	34.80
	Requires assistance	272	65.20
Duration of stroke	< 1 year	315	75.54
	1–3 years	70	16.79
	4–6 years	14	3.36
	> 6 years	18	4.32
Interoceptive sensibility		90.46 ± 44.92	
Health literacy		69.33 ± 22.82	

Family monthly income per capita was categorized as low (< 1,500 RMB), moderate (1,500–3,000 RMB), or high (> 3,000 RMB), based on local income levels in Zunyi, Guizhou.

### 3.2 Latent class analysis of psychological distress

**Table 2** presents the comparison of fitting indicators for the 1 to 5 category models in the latent class analysis (LCA) of psychological distress in 417 patients. The results show that as the number of categories increases, the log-likelihood value gradually rises, but the trends of information criteria such as AIC and BIC are not consistent: BIC reaches its minimum value at the 3-category model (C3) (2590.33), and then the BIC of the 4-category and 5-category models increase significantly; the LMR test shows that the 3-category model is significantly better than the 2-category model (p < 0.001), while the improvement of the 4-category compared to the 3-category is only marginally significant (p = 0.017), and the 5-category compared to the 4-category has no significant improvement (p = 0.173); BLRT is not significant at the 5-category model (p = 0.208). In addition, although the AIC and aBIC of the 4-category and 5-category models slightly decrease, the decrease from C3 to C4 and C5 is significantly reduced, and the Entropy value in the 3-category model has reached 0.92, indicating good classification quality. Based on the principle of minimum BIC, significant changes in LMR and BLRT, the model’s simplicity, and the interpretability of the classification, the final choice is to select 3 categories as the optimal model for describing the heterogeneity in patients’ psychological distress. **Table 2** presents the model fit indices for all tested class solutions. The 4th class comprised less than 5% of the sample and did not represent a clinically distinct profile. Therefore, the 3-class model was retained for its parsimony and interpretability. Additionally, the average probabilities of stroke patients being assigned to each class ranged from 94.5% to 97.2%, which underscores the robustness and reliability of the three-class model, as detailed in **Table 3**.

**Table 2:** Model fit indices for the latent class analysis of patients’ psychological distress (n=417).

Model	k	Log (L)	AIC	BIC	aBIC	Entropy	LMR	BLRT
C1	9	-2287.68	4593.37	4629.66	4601.10			
C2	19	-1332.05	2702.10	2778.73	2718.43	0.98	<0.001	<0.001
C3	29	-1207.69	2473.37	2590.33	2498.30	0.92	<0.001	<0.001
C4	39	-1185.50	2449.00	2606.29	2482.54	0.93	0.017	<0.001
C5	49	-1175.11	2448.22	2645.84	2490.35	0.93	0.173	0.208

k, Number of classes in the model; Log(L), Log-likelihood value; AIC, Akaike Information Criterion; BIC, Bayesian Information Criterion; aBIC, Adjusted Bayesian Information Criterion; Entropy, A measure of classification uncertainty; LMR, Lo-Mendell-Rubin Test; BLRT, Bootstrapped Likelihood Ratio Test.

**Table 3:** Average membership probabilities for each latent class of participants (n=417).

Class	C1 (%)	C2 (%)	C3 (%)
C1	94.5	1.6	3.9
C2	1.2	98.8	0.0
C3	2.8	0.0	97.2

**Figure 1:**
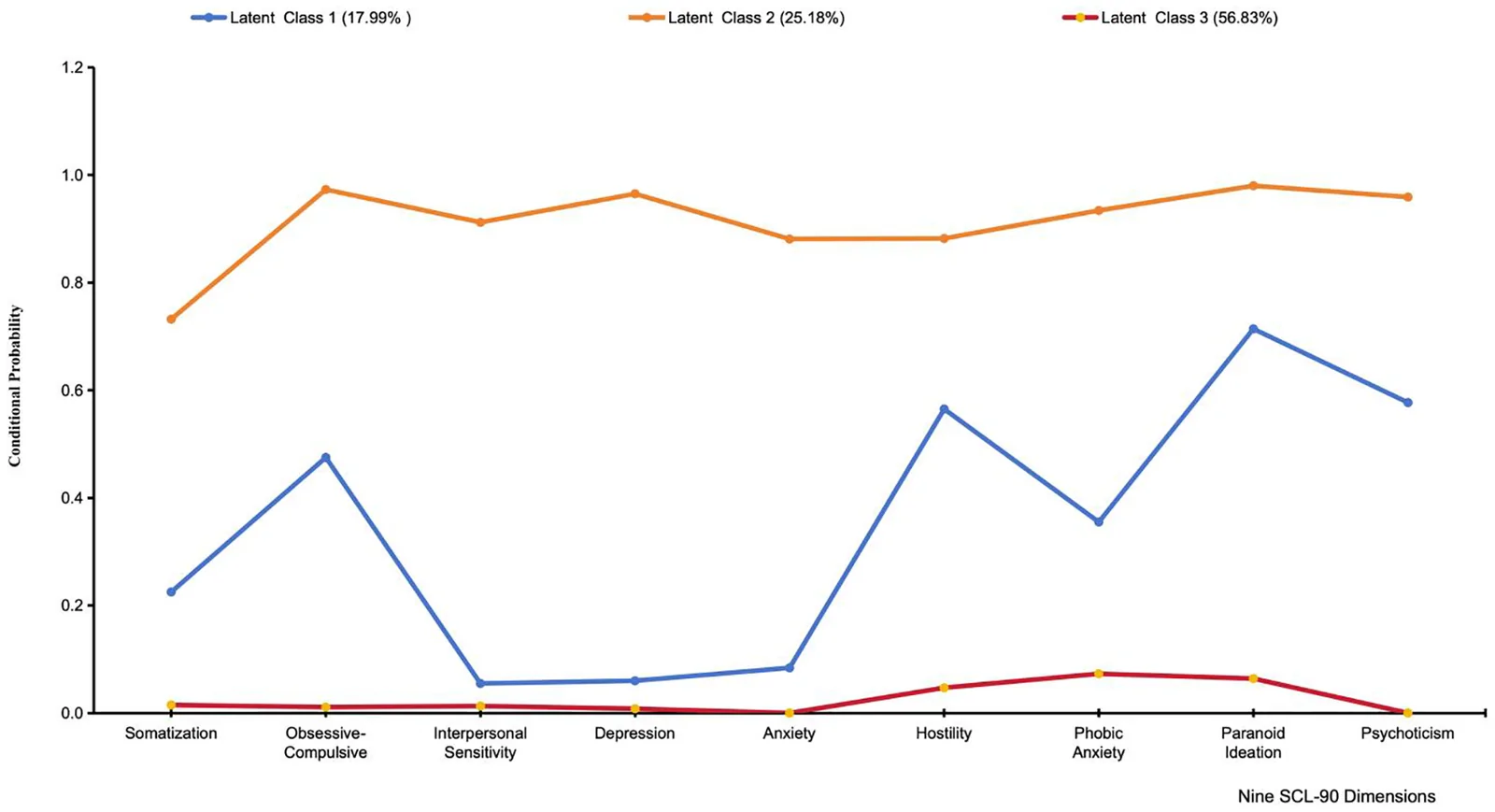
Conditional probabilities of SCL-90 symptom dimensions across latent class.

**Figure 1** presents a visual depiction of the three distinct classes identified among stroke patients. In this graph, the x-axis denotes the nine SCL-90 dimensions (somatization, obsessive-compulsive, interpersonal sensitivity, depression, anxiety, hostility, phobic anxiety, paranoid ideation, and psychoticism), while the y-axis indicates the conditional probability of reporting each symptom dimension within each class. Class 1, identified as the “High Paranoid Ideation and Hostility group” (comprising 17.99% of the sample), is characterized by stroke patients exhibiting the highest levels of Paranoid Ideation and a relatively elevated risk of Hostility. Class 2, labeled the “Generalized Psychological Distress group” (25.18% of the sample), consists of stroke patients with a broad range of psychological distress risks. This group has the highest scores across all nine dimensions of psychological distress and represents the second largest subgroup. Class 3, termed the “Low Psychological Distress group” (56.83% of the sample), includes stroke patients with the lowest levels of psychological distress risk. This group also has the lowest scores in all nine dimensions of psychological distress and is the largest subgroup among the three classes.

### 3.3 Factors associated with latent class membership

Univariate analysis revealed that the three latent classes were influenced by several factors, including gender, duration of stroke, interoceptive sensibility, and health literacy, all of which were statistically significant (p < 0.05). These findings are detailed in **Table 4**.

**Table 4:** Demographic and characteristics by latent class (n=417).

Variables	Categories	Class 1 (n=75)	Class 2 (n=105)	Class 3 (n=237)	F/X/H	*P*
Age, Mean (SD)		61.49 ± 13.88	59.40 ± 12.93	62.86 ± 11.26	2.960	0.053
Gender	Male	37 (49.33)	95 (90.48)	128 (54.01)	47.822	<0.001
	Female	38 (50.67)	10 (9.52)	109 (45.99)		
Residence	Countryside	67 (89.33)	94 (89.52)	211 (89.03)	0.020	0.990
	City	8 (10.67)	11 (10.48)	26 (10.97)		
Ethnic group	Han-Nationality	67 (89.33)	98 (93.33)	222 (93.67)	1.664	0.435
	Ethnic minority	8 (10.67)	7 (6.67)	15 (6.33)		
Education	Elementary	51 (68.00)	75 (71.43)	170 (71.73)	6.274	0.393
	Middle school	21 (28.00)	20 (19.05)	46 (19.41)		
	High school	3 (4.00)	5 (4.76)	14 (5.91)		
	College	0 (0.00)	5 (4.76)	7 (2.95)		
Marital status	Married	73 (97.33)	99 (94.29)	228 (96.20)	1.149	0.563
	Widowed/divorced	2 (2.67)	6 (5.71)	9 (3.80)		
Living Situation	Living alone	0 (0.00)	4 (3.81)	18 (7.59)	7.897	0.095
	Living with spouse	64 (85.33)	82 (78.10)	178 (75.11)		
	Living with children or grandchildren	11 (14.67)	19 (18.10)	41 (17.30)		
Family monthly income	< 1,500 RMB	25 (33.33)	27 (25.71)	71 (29.96)	2.913	0.572
	1,500–3,000 RMB	44 (58.67)	62 (59.05)	139 (58.65)		
	> 3,000 RMB	6 (8.00)	16 (15.24)	27 (11.39)		
Types of stroke	Ischemic stroke	46 (61.33)	76 (72.38)	165 (69.62)	2.650	0.267
	Hemorrhagic stroke	29 (38.67)	29 (27.62)	72 (30.38)		
Self-care capability	Independent	26 (34.67)	35 (33.33)	83 (35.02)	0.092	0.955
	Requires assistance	49 (65.33)	70 (66.67)	154 (64.98)		
Duration of stroke	< 1 year	56 (74.67)	95 (90.48)	164 (69.20)	18.023	0.000
	1–3 years	12 (16.00)	8 (7.62)	50 (21.10)		
	4–6 years	3 (4.00)	1 (0.95)	10 (4.22)		
	> 6 years	4 (5.33)	1 (0.95)	13 (5.49)		
Interoceptive sensibility, Mean (SD)		88.43 ± 33.30	79.32 ± 43.87	96.03 ± 46.50	5.230	0.006
Health literacy		69.83 ± 21.02	74.97 ± 26.61	66.68 ± 21.13	4.908	0.008

Class 1, High Paranoid Ideation and Hostility group; Class 2, Generalized Psychological Distress group; Class 3, Low Psychological Distress group. Values in parentheses are percentages. Family monthly income per capita was categorized as low (< 1,500 RMB), moderate (1,500–3,000 RMB), or high (> 3,000 RMB), based on local income levels in Zunyi, Guizhou.

To explore the factors linked to psychological distress, multiple logistic regression analyses were performed, with Class 3 serving as the reference group. The findings, detailed in **Table 5**, revealed that health literacy and interoceptive sensibility mean scores were significantly related to membership in Classes 1 and 2. Specifically, the odds ratios (OR) and 95% confidence intervals (CI) were as follows: for Class 1, OR = 1.019 (95% CI: 1.003–1.036) and OR = 1.057 (95% CI: 1.039–1.075); for Class 2, OR = 0.988 (95% CI: 0.980–0.997) and OR = 0.974 (95% CI: 0.965–0.983). Additionally, male patients and those with a stroke duration of less than one year were significantly more likely to be in Class 2, with OR values of 9.281 (95% CI: 4.347–19.815) and 23.594 (95% CI: 2.592–214.62), respectively.

**Table 5:** Multinomial logistic regression results of latent class (n=417).

Variables	Class1 *vs* Class3					Class2 *vs* Class3				
	B	OR	Std. error	Sig.	95% CI	B	OR	Std. error	Sig.	95% CI
Intercept	-1.492	0.225	0.793	0.060		-6.875	0.001	1.313	<0.001	
Health literacy	0.019	1.019	0.008	0.019	(1.003,1.036)	0.055	1.057	0.009	<0.001	(1.039,1.075)
Interoceptive sensibility	-0.012	0.988	0.004	0.006	(0.98,0.997)	-0.026	0.974	0.005	<0.001	(0.965,0.983)
Gender (Ref: Female)										
Male	-0.207	0.813	0.286	0.469	(0.464,1.423)	2.228	9.281	0.387	<0.001	(4.347,19.815)
Duration of stroke (Ref: > 6 years)										
< 1 year	0.343	1.409	0.610	0.574	(0.426,4.663)	3.161	23.594	1.127	0.005	(2.592,214.62)
1–3 years	-0.129	0.879	0.669	0.847	(0.237,3.263)	1.339	3.815	1.181	0.257	(0.377,38.612)
4–6 years	0.187	1.206	0.890	0.834	(0.211,6.905)	0.763	2.145	1.579	0.629	(0.097,47.355)

Class 1, High Paranoid Ideation and Hostility group; Class 2, Generalized Psychological Distress group; Class 3, Low Psychological Distress group (reference). OR, odds ratio; SE, standard error; CI, confidence interval.

## 4 Discussion

This study identified distinct latent classes of psychological distress among stroke patients and explored the associated influencing factors. Three latent classes were determined in our study: Class 1, labeled as the “High Paranoid Ideation and Hostility group,” accounted for 17.99% of the participants; Class 2, labeled as the “Generalized Psychological Distress group,” comprised 25.18% of the participants; and Class 3, labeled as the “Low Psychological Distress group,” made up 56.83% of the participants. This classification highlights the heterogeneity of psychological distress among stroke patients, which is crucial for understanding their diverse needs and developing targeted interventions. Among these, the “high paranoia and hostility group” accounted for 17.99%. This group exhibited pronounced paranoia and hostility, which may indicate underlying anxiety and distrust. These symptoms can severely impair patients’ ability to engage in rehabilitation and social interactions (19). Previous studies have shown that such psychological states can lead to increased stress and reduced quality of life (26). Therefore, targeted psychological interventions are necessary to address these issues. The “generalized psychological distress group” accounted for 25.18%. This type of psychological distress is relatively common among stroke patients and can significantly hinder their rehabilitation progress. Research indicates that comprehensive psychological support, including social support and counseling, can improve the mental health of stroke patients (26). Future interventions should focus on providing a supportive environment for this group and addressing the multifaceted nature of psychological distress. The “low psychological distress group” accounts for 56.83%. This group exhibits minimal psychological distress, suggesting they may have better coping mechanisms or social support systems. However, it is important to monitor this group for any emerging signs of psychological issues, as stroke may have long-term psychological impacts that may not manifest immediately.

Health literacy emerged as a significant predictor of psychological distress in our stroke sample. Rather than conferring protection, higher literacy scores increased the odds of belonging to Class 1 (OR = 1.019) and Class 2 (OR = 1.057) relative to Class 3.A “knowledge burden” may explain this unexpected pattern. Patients who more clearly grasp their prognosis, recurrence risk, and long-term disability may grow more anxious and hypervigilant a dynamic similar to cyberchondria, where greater health information literacy amplifies rather than reduces anxiety through excessive information seeking (27–29). This interpretation remains tentative and requires longitudinal validation. Unmeasured factors such as pre-morbid personality traits and social support may also influence the observed association.

This study found that interoceptive sensibility is an important factor associated with psychological distress in stroke patients. Specifically, compared with the “low psychological distress group” (Category 3), members of the “high paranoid beliefs and hostility group” (Category 1) and the “generalized psychological distress group” (Category 2) showed a negative correlation with lower average interoceptive sensibility scores. This finding is consistent with previous research, which has shown that lower internal sensitivity is associated with higher levels of psychological distress (30). The negative correlation between interoceptive sensibility and psychological distress can be attributed to multiple mechanisms. Interoceptive sensibility refers to the awareness and perception of internal bodily sensations, which are closely linked to emotional regulation and stress responses. Individuals with lower interoceptive sensibility may struggle to identify and interpret their internal bodily signals, leading to impaired emotional regulation and increased vulnerability to psychological distress (31). For example, research suggests that individuals with lower interoceptive sensibility are more likely to experience anxiety and depression because they may be unable to effectively regulate their emotional responses to stressors (32). Additionally, lower interoceptive sensibility may impair an individual’s ability to engage in adaptive coping strategies. When individuals have weaker awareness of their internal states, they may be less likely to recognize the need for self-care or seek appropriate support, thereby further exacerbating their psychological distress. This is supported by research indicating that interoceptive sensibility is associated with better emotional regulation and lower levels of anxiety and depression (32). Our findings suggest that interoceptive sensibility is associated with psychological distress, which may inform future intervention research aimed at improving psychological outcomes in stroke patients.

Our study revealed that male stroke patients and those with a stroke duration of less than one year were significantly more likely to belong to Class 2. This finding is in line with previous studies that have shown a higher prevalence of psychological distress among male stroke patients and those in the early stages of recovery. Several factors may contribute to this phenomenon (33). Firstly, males are more likely to have severe stroke symptoms and a higher prevalence of traditional risk factors, such as smoking and alcohol consumption. These factors can exacerbate psychological distress (34). Additionally, the first year after a stroke is a critical period for recovery and adjustment. Patients with a stroke duration of less than one year may still be in the acute phase of their illness, experiencing significant physical and emotional challenges. This period is often marked by uncertainty and a higher burden of care, which can intensify psychological distress. Moreover, gender differences in coping mechanisms and social support may play a role. Males may be less likely to seek help or express their emotional needs, leading to increased psychological distress (35). In contrast, females may have better access to social support networks, which can mitigate the impact of stroke-related stressors (33). Our results underscore the need for targeted interventions for male stroke patients and those in the early stages of recovery. Future research should focus on developing gender-specific and time-sensitive interventions to address the unique needs of these patient groups. Additionally, further studies should explore the underlying mechanisms linking gender, stroke duration, and psychological distress to inform more effective treatment strategies.

## 5 Limitations

This research acknowledges several limitations. First, participants were recruited from a single tertiary hospital’s cerebrovascular ward in China using convenience sampling, limiting the generalizability of our findings. Second, data collection relied solely on self-reported information from patients, which may introduce response bias, such as social desirability or recall bias. Third, because this is a cross-sectional study, it can identify correlations between variables but cannot establish causality. Fourth, although latent class analysis was used for grouping, this method involves some subjectivity, and the resulting classifications should be interpreted cautiously, as they may not be definitive. Fifth, the wide confidence intervals for stroke duration reflect small sample sizes in some categories, and these results should be interpreted cautiously. Future research should employ multi-center longitudinal designs with larger, more diverse samples to validate our findings and examine the stability of latent classes over time. In addition, using objective measures alongside self-reported data could help reduce response bias and provide a more comprehensive assessment of psychological distress. Future studies may also explore additional psychosocial and biological factors, such as social support, coping styles, and inflammatory markers, to better understand the mechanisms underlying psychological distress in stroke patients. Furthermore, intervention studies are needed to test whether the classification identified in this study can effectively guide personalized psychological interventions and improve patient outcomes.

## 6 Conclusion

In the current study, we identified three latent classes of psychological distress among stroke patients, highlighting its heterogeneity. Moreover, our findings indicated that four main factors (health literacy, interoceptive sensibility, gender, and duration of stroke) were significantly associated with psychological distress. Adopting a personalized approach that addresses the unique needs and psychological traits of patients, rather than relying on a standardized model, may improve patient adherence and make more efficient use of medical and social resources. The classification provided by this study may inform the development of targeted interventions and serves as a reference for future multi-center, large-sample studies.

## Data Availability

The original contributions presented in the study are included in the article/supplementary material. Further inquiries can be directed to the corresponding author.
